# Measurements of Vitamin D and inflammation factors in a psychiatric outpatient clinic

**DOI:** 10.1192/j.eurpsy.2025.2029

**Published:** 2025-08-26

**Authors:** S. Kocijancic Azzaoui, P. Pregelj

**Affiliations:** 1 Psycho-oncology, Institute of Oncology; 2 Center of mental health, University psychiatric clinic Ljubljana, Ljubljana, Slovenia

## Abstract

**Introduction:**

Vitamin D is a fat-soluble vitamin that together with parathyroid hormone (PTH) regulates blood calcium and phosphorus levels. Vitamin D also has anti-inflammatory, antioxidant, and neurotrophic properties. It acts through the vitamin D receptor (VDR), which has been found throughout the body, including the nervous system. Its deficiency is associated with various diseases, including depression and schizophrenia. It is estimated that approximately 1 billion people worldwide have vitamin D deficiency, while 50% of people worldwide (4 billion people) are thought to have insufficient levels of vitamin D.

**Objectives:**

The retrospective study aimed to determine whether routine laboratory tests in a psychiatric office/outpatient clinic can find a connection between vitamin D levels and inflammatory parameters such as CRP, leukocytes, and the neutrophil-to-lymphocyte ratio (NLR).

**Methods:**

Data was collected from a psychiatric office/outpatient clinic between the years 2020 and 2023. We included the patients (and their basic data, such as gender, age, diagnosis during treatment) whose laboratory results had vitamin D levels as well as a complete blood count (leukocytes, neutrophils, and lymphocytes) and c-reactive protein (CRP). We calculated the ratio of neutrophils to lymphocytes (NLR) and performed Spearman’s correlation, where a p-value of <0.05 indicated a statistically significant change.

**Results:**

Between 2020 and 2023, 88 laboratory tests were conducted in the outpatient office, that included vitamin D levels, of these, 67 had all the necessary data. Vitamin D deficiency was present in 65% of patients. We found that CRP was not sensitive enough for our study, as 83% of CRP values were below 8 mg/L. After performing a correlation between vitamin D levels and leukocytes and NLR, we did not find a statistically significant connection.

**Conclusions:**

We did not find statistically significant corelation between the level of vitamin D and NLR and leucocytes. We would recommend further research that uses a larger sample of patients and conduct measurements during acute illness or exacerbation to obtain more reliable conclusions.

**Disclosure of Interest:**

None Declared

